# Botanical Pesticides: Role of *Ricinus communis* in Managing *Bactrocera zonata* (Tephritidae: Diptera)

**DOI:** 10.3390/insects15120959

**Published:** 2024-12-02

**Authors:** Rasheed Akbar, Sadia Manzoor, Rashid Azad, Gul Makai, Junaid Rahim, Umer Ayyaz Aslam Sheikh, Amjad Ali, Tariq Aziz, Hafiz Ishfaq Ahmad, Mukhtar Ahmed, Daolin Du, Jianfan Sun

**Affiliations:** 1 Institute of Environment and Ecology, School of Environment and Safety Engineering, Jiangsu University, Zhenjiang 212013, China; rasheed.akbar@uoh.edu.pk; 2 Department of Entomology, Faculty of Physical and Applied Sciences, The University of Haripur, Haripur 22062, Pakistan; 3 Department of Zoology, Sardar Bahadur Khan Women’s University, Quetta 86400, Balochistan, Pakistan; 4 Department of Entomology, Faculty of Agriculture, University of Poonch, Rawalakot 12350, Azad Jammu and Kashmir, Pakistan; 5 School of Material Science & Engineering, Jiangsu University, Zhenjiang 212013, China; 6 Faculty of Civil Engineering and Mechanics, Jiangsu University, Zhenjiang 212013, China; 7 Department of Animal Breeding and Genetics, Faculty of Veterinary and Animal Sciences, The Islamia University of Bahawalpur, Bahawalpur 63100, Pakistan; 8 Department of Zoology, College of Science, King Saud University, P.O. Box 2455, Riyadh 11451, Saudi Arabia; mahmed1@ksu.edu.sa; 9 Jingjiang College, Jiangsu University, Zhenjiang 212013, China; 10 Jiangsu Collaborative Innovation Center of Technology and Material of Water Treatment, Suzhou University of Science and Technology, Suzhou 215009, China

**Keywords:** *Bactrocera zonata*, *Ricinus communis*, isolation, phytochemistry and purification

## Abstract

The melon fruit fly, *Bactrocera zonata* (Coquillett) (Diptera: Tephritidae), is a notorious pest, posing a significant threat to a wide range of fruits and vegetables, leading to substantial agricultural losses worldwide. With growing concerns over chemical pesticide resistance and environmental safety, plant-based insecticides have emerged as eco-friendly and economically sustainable alternatives. In this context, the present study delves into the insecticidal potential of *Ricinus communis* extracts against *B. zonata*. The crude extract of *R. communis* was systematically fractionated using a series of organic solvents with increasing polarities. The fraction demonstrating the highest insecticidal activity was further purified for the isolation of bioactive compounds, employing advanced chromatographic techniques such as Column Chromatography, coupled with state-of-the-art analytical methods including Gas Chromatography-Mass Spectrometry (GC-MS) and Fourier Transform Infrared Spectroscopy (FTIR). Bioassays were conducted on *B. zonata* using the crude extract and its fractions in n-hexane, methanol, and ethyl acetate. Among the isolated compounds, 11,14,17-Eicosatrienoic acid was identified in both the methanol and ethyl acetate fractions. This compound exhibited remarkable insecticidal efficacy, with an LC_50_ value of 1.36%, a linearity of R^2^ = 0.64, and a statistically significant probability (*p* < 0.01). Particularly, 11,14,17-Eicosatrienoic acid emerged as the most potent bioactive agent against *B. zonata* highlighting its potential as a natural insecticide. These findings underscore the potential of *R. communis* as a valuable source of bioactive compounds for the sustainable management of *B. cucurbitae*. This study not only broadens the scope of plant-based pest control strategies but also opens avenues for further exploration of natural compounds in integrated pest management.

## Simple Summary

The melon fruit fly, *Bactrocera zonata*, is a major pest affecting various fruits and vegetables, causing severe agricultural losses globally. With rising concerns over pesticide resistance and environmental impacts, plant-based insecticides have become promising sustainable alternatives. This study investigates the insecticidal potential of *Ricinus communis* extracts against *B. zonata*. Sequential fractionation of the crude extract using solvents with increasing polarities led to the isolation of bioactive compounds, with 11,14,17-Eicosatrienoic acid identified in methanol and ethyl acetate fractions. This compound showed significant insecticidal activity, with an LC_50_ of 1.36% and high statistical significance (*p* < 0.01). These findings highlight *R. communis* as a promising natural source for pest management, promoting eco-friendly pest control solutions.

## 1. Introduction

Fruit flies, belonging to the order Diptera and the family Tephritidae, consist of four thousand and five hundred species, posing a significant threat to agricultural production. The two polyphagous fruit flies currently established in different regions of Pakistan are the peach fruit fly, *Bactrocera zonata* (Saunders), and the cucurbit fruit fly, *Bactrocera cucurbitae* (Coquillett). These species are the most dangerous and extensively distributed pests throughout the country [1]. The economic value of fruits and vegetables may eventually suffer losses due to severe attacks from these pests. These pests adapt to the various climates found all over the world. They are mostly found in tropical and subtropical regions of the world, which causes significant economic losses and increases the risk of them spreading to other regions. Soft-bodied fruits and vegetables like mangoes, peaches, guava, oranges, bananas, pumpkins, and bitter guard are the main targets of these pests’ attacks. According to reports, more than 70 species of *Bactrocera* are considered to be serious crop pests worldwide [2].

Both *B. zonata* and the oriental fruit fly *B. dorsalis* are alien invasive pests of horticultural crops known to be native to Asia and infest a variety of host fruits and vegetables [3]. More than 50 host plants, including guava, mango, peach, papaya, orange, and grapefruit (edible hosts), as well as jujube and ivy gourd (wild hosts), have been recorded to be attacked by these pests [4]. The life cycle of *B. zonata* can be recognized by the insertion of eggs into fruit tissues and the feeding of larvae inside the pulp. As a result, different stages of the fruit are susceptible to the effects of pesticides, and fruits that are in the early stages of contamination do not exhibit obvious appearance symptoms that would point to damage or deterioration. Fruits that have external damage from pests may sustain punctures, feeding marks, or other wounds. These damages change the fruit’s surface features, which have an impact on its reflectance qualities [5].

To overcome the issues caused by these pests, it is necessary to explore environmentally friendly and sustainable strategies for the management of *B. zonata*. *Ricinus communis* L. (Euphorbiaceae), due to its insecticidal properties, is the best option for controlling *B. zonata* [6]. According to Franke et al. [7], *R. communis* is a very adoptable plant with a wide range of applications. Its 83 bioactive chemicals, including alkaloids, terpenoids, flavonoids, derivatives of benzoic acid, coumarins, and fatty acids, have been found in different portions of the plant by previous research [8,9]. These substances highlight a wide range of pharmacological activities. According to Akbar et al. [10,11], the plant extracts showed potential in reducing insect infestations and providing a more environmentally friendly method for the management of insect pests. The increase in infestation or revival of some insect species due to the loss of their natural enemies are some of the limitations of synthetic pesticides. Further disadvantages include the development of insect resistance and excessive pesticide residues in fruits and vegetables and the environment.

This study aims to comprehensively evaluate the insecticidal potential of bioactive compounds isolated and characterized from the leaves of *R. communis*. Our focus is on determining their effectiveness against *B. zonata*, a significant agricultural pest. By exploring the pesticidal properties of these naturally derived substances, we seek to contribute valuable insights for sustainable pest management strategies. We will be able to identify the most effective extracts by using various solvents, including methanol, ethyl acetate, and n-hexane, in the extraction procedure. The identification and characterization of the particular bioactive substances accountable for the indicated insecticidal actions will be aided by Gas Chromatography-Mass Spectrometry (GCMS) study. For the management of fruit fly populations, the farmers mostly used synthetic insecticides [12]. Changing the pest status from minor to major, ecological disruption, and health risks to farmers are other significant concerns [13]. It is therefore essential to investigate alternative plant-based pesticides.

## 2. Materials and Methods

### 2.1. Plant Sample Preparations

The leaves of *R. communis* were collected from various locations of district Haripur Khyber Pakhtunkhwa Pakistan. The leaves were then left to air-dry at room temperature for one week, after which they were ground into a fine powder. Following the extraction methodologies by [14] with some minor modifications, we took 12 g of dried plant powders and mixed it in 120 mL of methanol, ethyl acetate, and n-hexane and put each mixture on orbital shaker (Biobase Meihua Trading, Jinan, China) at 160 rpm (revolutions per minute) for 60 min. After that, we placed the mixture at room temperature for 24 h and then it was filtered by Whatman no. 1 filter paper. For accuracy, the filtrations were repeated three times for maximum filtrates. The filtrates were placed in rotary for further concentration. After that, the stock solutions, i.e., 10%, were covered by micro film sheaths and stored at room temperature for further experimentations. The stock solutions of methanol, ethyl acetate, and n-hexane were further diluted to make 0.5%, 1%, 1.5%, and 2%. The same dilutions were made for the three types of solvents.

### 2.2. B. zonata Collection and Rearing

For the collection of *B. zonata*, we collected different fruits from the main supermarket, as well as infested fruits from the orchards, and put the fruits in jars by making some modifications to the jars. We took a plastic jar with dimensions of 2 × 2 cm and filled 1/3rd of the bottom of the jar with very fine sand. We placed the infested vegetables on fine sand to allow larvae emerging from the host to burrow into the sand and pupate. Once pupation occurred, we collected them and secured the jar with a nylon cloth to prevent the entry of other insects and ensure adequate aeration. We continuously revised the experiment in order to collect the adult third generation, which is completely pure. We provided a mixture of yeast, sugar and water, which was placed within a petri dish as a dietary supplement in the cage.

### 2.3. Toxicity of Plant Extracts Against B. zonata

The toxicity of three different extracts of *R. communis* were tested against *B. zonata*. Each solvent was further diluted to make it 0.5%, 1.0%, 1.5%, and 2.0%. For the control, we used pure methanol, ethyl acetate, and n-hexane. The insects were kept in test containers without any supplementary food or water for the duration of the bioassays to ensure that the observed effects were solely attributable to the treatments. This approach allowed for the accurate assessment of the extracts’ toxicity. *B. zonata* adults are known for their swift flight capabilities, which can pose challenges during handling. To manage this, we briefly chilled the adults at −2 °C for one minute, effectively immobilizing them without causing harm. Using a fine camel-hair brush, we then gently transferred each chilled insect into petri dishes, each measuring 9 cm in diameter, ensuring minimal stress and optimal conditions for subsequent experiments. We applied 1.00 μL solutions of each solvent of four different concentrations on the dorsal surface of the insect using a microliter syringe (Hamilton 700, Bonaduz, Switzerland). There were ten *B. zonata* in each treatment. After 24, 48, and 72 h exposure periods, the dead *B. zonata* were calculated and converted into percentage mortalities. There were three replications and the experiments were carried out in completely randomized design (CRD). The following equation was used to find the corrected mortalities:

(1) $$Corrected\;mortalities=\frac{\%Nt-\%Nc}{100-\%Nc}\times 100$$

where *Nt* is the mortalities in treatment, and *Nc* is the mortalities in control.

### 2.4. Preparation of Plant Extracts for Chromatography

Solid liquid extraction method was used for the extraction of plant materials. Methanol, ethyl acetate, and n-hexane were carefully chosen and used to ensure effective extraction and reliable results. We dissolved 30 g of plant fine powders in 150 mL of methanol in 500 mL beaker and put the beaker for two hours at 150 rpm on orbital shaker. We placed the mixture at room temperature for 72 h. We used the Whatman no. 1 filter paper for filtrations. Conical flasks were used to collect the filtrates as stock solutions. This method was revised for other solvents including ethyl acetate and n-hexane. After extracting with methanol, ethyl acetate, and n-hexane, we removed extra solvent using a rotary evaporator under vacuum conditions at 20 °C, resulting in a thick, semi-solid mass. After rotary, the extracts were dried and put in the glass petri dishes covered with aluminum foil and stored at room temperature.

### 2.5. Column Chromatography

For gravitational column chromatography, a glass column measuring 600 mm × 15 mm with a stopper at the bottom was used. To prevent the silica gel from escaping, a frit (disc) was fitted at the column’s base. Silica gel (60, 100–200 mesh size, Merck, Karachi, Pakistan) served as the stationary phase. The column was packed using the dry packing method, ensuring that no air bubbles were trapped during the addition of silica gel. A 0.5 cm layer of fine sand was applied on top of the settled silica gel to further support the packing.

The crude mixture of compounds was dissolved in the chosen eluent and carefully loaded onto the pre-packed column using a Pasteur pipette. The elution process began by closing the stopcock for 24 h to allow the mixture to settle under gravity. After this period, the stopcock was opened to let the mobile phase (solvent) flow through the column until the solvent reached the top layer of sand. Gradient elution was employed using solvents of varying polarities: for the n-hexane extract, pure n-hexane was followed by ethyl acetate and methanol, while for the methanol extract, pure methanol was used first, followed by ethyl acetate and methanol. This gradient elution effectively separated the compounds based on their interactions with the mobile and stationary phases, with polar compounds eluting with polar solvents and non-polar compounds with non-polar solvents. Fractions were collected every 30 min in small test tubes at a flow rate of 20–27 mL per 30 min. To concentrate the collected fractions, a rotary evaporator was used to remove excess solvent. The insecticidal activity of each fraction was tested, and FTIR (Fourier Transform Infrared Spectroscopy, Thermo Fisher Scientific China, Shanghai, China) and GC-MS (Gas Chromatography-Mass Spectrometry, PerkinElmer Shanghai, China) analyses were performed to identify the compounds responsible for toxicity.

### 2.6. FTIR Analysis

Thermo Nicolet 380 FT-IR Spectrophotometer was used for analysis of compounds. It was operated using OMINIC version 7.3 controls and processing software from Thermo Electron Corporation (Seoul, Republic of Korea), using Sodium Chloride discs. Absorption bands were coated in wave numbers (cm^−1^). Isolated compounds were dissolved in chloroform and mixed with nujol mull for their analysis

### 2.7. GC-MS Analysis

A GC (Agilent technologies 5890, PerkinElmer, Shanghai, China) with helium as carrier gas was utilized along with electron spray ionization connected to autosampler injection system. The analysis was accomplished using the Acquired Method Default, with temperatures ranging from 40 to 340 °C over 28 min.

### 2.8. Insecticidal Activity of Collected Fractions

To check the toxicity effects of collected fractions, direct toxicity test was done. The adults of *B. zonata* were chilled for a period of 30 s at −2 °C. After that, the *B. zonata* movements were stopped, then with the help of camel brush, the tested insects were transferred into petri dishes with size of 9 cm in diameter. One microliter (1.00 μL) of the solution from each fraction was precisely applied to the dorsal surface of the thorax using a Hamilton 700 microliter syringe (Bonaduz, Switzerland), ensuring accurate dosage for subsequent analysis. In each replication along with control, we used ten *B. zonata* per treatment. In control, we applied only distilled water. The percentage mortalities were calculated after 24, 48, and 72 h’ exposure period. This method was applied for other fractions also and the experiments were carried out in completely randomized design (CRD) with three replications.

### 2.9. Data Analyses

The data were examined using analysis of variance (ANOVA), where significant variations between the treatments were examined. Mean values were then significantly separated by using least significant difference (LSD) test at a 5% level. The percentage mortalities were corrected using Abbott’s formula [15]. After that, the percentage mortality in adult *B. cucurbitae* was analyzed using the Log-Probit model to calculate the 50% and 90% lethal concentrations (LC_50_/LC_90_) [16]. The Statistical Package for the Social Sciences (SPSS) version 20 was used to perform the Probit analysis and analysis of variance. The extracts obtained by methanol, ethyl acetate, and n-hexane were compared by employing two-way hierarchical cluster analysis (HCA). The cluster analysis was performed by complete linkage method and the relationship among the extraction solvents and the organic compounds was measured as Euclidean distance. The intensity of each organic compound with respect to the organic solvents was compared by heat map diagram. The cluster analysis and the heat map analysis were performed by following [17], using an open-source online resource www.bioinformatics.com.cn (accessed on 5 December 2023). Subsequently, the concentrations of the commonly occurring compounds in different solvent extracts were determined by multiplying the percentage composition of each organic compound with the dose of respective organic extract. Later, the relationship between the concentrations of bioactive compounds was established with the mortality data by employing linear regression analysis. The significance of the relationship was determined at 95% level of significance (*p* < 0.05). The analysis was performed by using Microsoft Excel 2022 (ANOVA).

## 3. Results

### 3.1. Ethyl Acetate Extract of R. communis Against B. zonata

The lethality of ethyl acetate extract from *R. communis* against adult *B. zonata* was assessed through direct exposure in jars at various concentrations over 24, 48, and 72 h, as shown in Table 1. The results indicated that the mortality in *B. zonata* was directly proportional to the concentration of the extract, with higher doses resulting in increased toxicity. The mortality rates in *B. zonata* were significantly higher at a 2% concentration, reaching 40.00%, while lower mortality rates were observed at a 0.5% concentration, with 13.00% mortality (df = 3, F = 3.47, *p* = 0.0910). *B. zonata* lethality was effected by the ethyl acetate extract of *R. communis* with an LC_50_ of 1.20% and an LC_90_ of 2.30% (slope = 1.106). The maximum mortalities of the ethyl acetate extract were 50.00% and the minimum concentrations exhibited 22.00% causalities in *B. zonata* after 48 h (df = 3, F = 7.47, *p* = 0.0189), which resulted in in an LC_50_ and LC_90_ of 4.20% and 61.41%, having a slope of 0.81. Higher mortalities in *B. zonata* were recorded at the 2.00% concentration, reaching 77.00%, and the least mortalities (30.00%) were recorded at 0.5% concentrations (df = 3, F = 16 and *p* = 0.029), which lead to an LC_50_ and LC_90_ of 1.60% and 10.31%, with a slope of 0.75 after the 72 h exposure period.

### 3.2. Toxic Potency of R. communis n-Hexane on B. zonata

After the direct exposure of adult *B. zonata* to *R. communis* extracts in n-hexane, mortality was found to increase with both concentration and duration of exposure (Table 2). At lower concentrations, i.e., 0.5%, the mortalities in *B. zonata* were 20.00% and at higher concentration 2% the mortalities 48.00% (df = 3, F = 3.47 and *p* = 0.0910) were observed. Thus, n-hexane extracts of *R. communis* were effective against *B. zonata* with LC_50_ and LC_90_ after 24 h of exposure of is 1.30% and 2.20% respectively with a slope of 1.23. After 48 h of application, at 0.5% concentration the lethality of *B. zonata* is 40.00% while at maximum i.e., 2% concentrations resulted in a lethality of 66.00% (df = 3, F = 33.58, *p* = 0.0004). The LC_50_ and LC_90_ values were 2.01% and 34.80%, respectively, with a slope of 1.08. Maximum mortalities to *B. zonata* were observed at 2% concentrations getting 86.60%, while the lowest 53.00% mortalities were recorded at 0.5% concentrations (df = 7, F = 16, *p* = 0.0219). LC_50_ 0.94% and LC_90_ 6.75%, respectively, with a slope of 0.42 after 72 h exposure period.

### 3.3. R. communis Methanol Extract Toxicity Against B. zonata

Extracts of *R. communis* in methanol caused significant mortalities in *B. zonata*, having LC_50_ and LC_90_ values of 5.72% and 67.09%, respectively, with a slope of 1.10 (Table 3). After contact exposure for 24 h, the mortality was directly proportional to the concentration of the extract. Exposure to the 0.5% methanol extract of *R. communis* was 20.00%, while mortality in those individuals exposed to the 2.0% extract was 46.00% (df = 3, F = 4.00 and *p* = 0.0701). Exposure to 0.5% for 48 h resulted in 31.00% mortality, while exposure to 2.0% killed 58.00% of the *B. zonata* (df = 3, F = 5.03 and *p* = 0.0446). This resulted in 48 h LC_50_ and LC_90_ values of 2.94% and 48.82%, with a slope of 0.80. After contact exposure to 0.5% for 72 h, mortality was 38.00%, while exposure to 2.0% caused mortality of 67.00% (df = 3, F = 6.05 and *p* = 0.0302). The 72 h LC_50_ and LC_90_ for the methanol *R. communis* extracts against *B. zonata* were 1.38% and 22.80%, respectively, with a slope of 0.889.

### 3.4. n-Hexane (A), Ethyl Acetate (B), and Methanol (C) R. communis Fraction Mortalities in B. zonata

**Figure 1:**
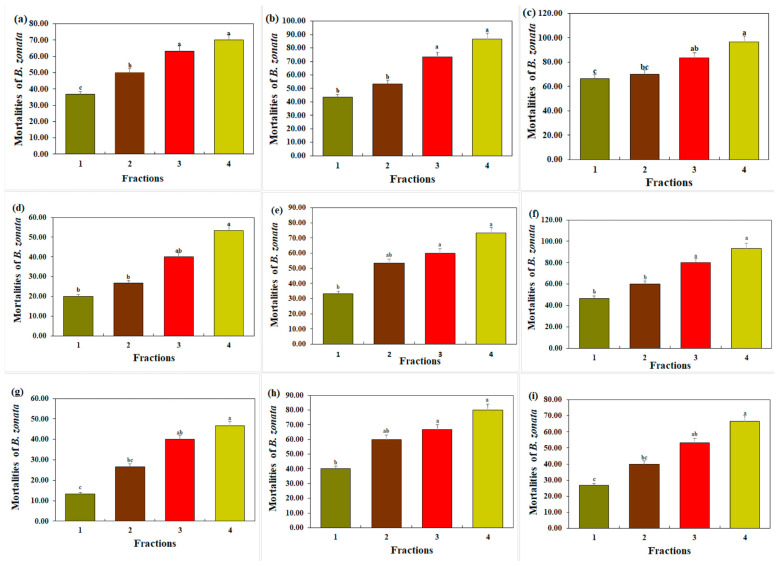
Mortality (%) in *B. zonata* exposed to *R. communis* extract fractions at different concentrations over time. X-axis represents concentration levels: 0.5% (labeled as 1), 1% (labeled as 2), 1.5% (labeled as 3), and 2% (labeled as 4). Panels (**a**–**c**) represent mortality after 24, 48, and 72 h of exposure to n-hexane fractions, respectively. Panels (**d**–**f**) illustrate results for ethyl acetate fractions at the same time intervals, while panels (**g**–**i**) show results for methanol fractions over 24, 48, and 72 h, respectively. Bars with different lowercase letters indicate that means are significantly different from each other at *p* = 0.05.

Mortalities in *B. zonata* increase with both the length and durations of time and concentrations of *R. communis* extracts when they are exposed (Figure 1). The highest mortality rate of 70.00% was seen at the highest concentrations, while the least lethality, 36.67%, was observed at a concentration of 0.5% after 24 h of exposure to the n-hexane fraction (A). The lower mortality of 43.33% was recorded at the 0.5% concentration, while the higher mortality of 86.67% was seen at the 2% concentration after 48 h (B) of exposure. Similarly, after 72 h of exposure (C), the mortalities were dose-dependent, with the highest value of 96.67%, while the mortality at 0.5%, which was the lowest concentration tested, was 66.67%.

When the *B. zonata* adults were exposed to 2% concentration extracts, a maximum mortality of 53.33% was observed, while the least lethality (33.33%) was observed when they were exposed to 0.5% extracts after 24 h of exposure to the ethyl acetate fraction (D). After 48 h of exposure (E), the highest mortality rate of 73.33% for *B. zonata* was exhibited at a 2% extract concentration, while the lower mortality rate of 33.33% was seen at a 0.5% concentration. Also, after 72 h of exposure (F), a mortality rate of 93.33% in *B. zonata* was observed at 2% extract concentrations, while a mortality rate of 46.67% was seen at 0.5% concentration.

Similarly, after 24 h of exposure to the methanol fraction in (Figure 1g), the highest mortality rate of 47% was observed at the higher concentrations, i.e., 2%, while the minimum mortality rate of 13.33% was seen at the 0.5% extract concentration. After 48 h of contact (H), the least lethality of 27% was seen at the 0.5% concentration and 67% mortality rate was seen at the 2% concentration. Similarly, after 72 h of exposure (I), the lethality was concentration-dependent and reached 80%, while the mortality when exposed to the lower concentrations of the 0.5% extract was 40%.

### 3.5. FTIR (Fourier Transform Infrared Spectroscopy) Analysis of Methanol, Ethyl Acetate, and n-Hexane Extract of R. communis

**Figure 2:**
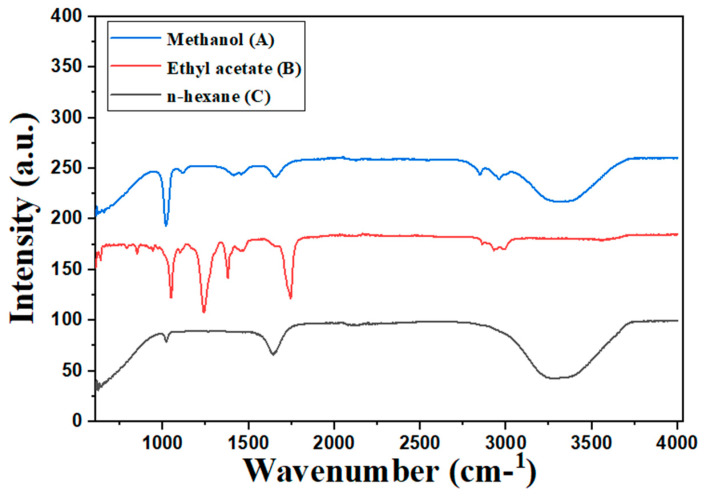
The comparative FTIR spectra of methanol (A), n-hexane (B), and ethyl acetate (C) extracts, illustrating distinct functional group absorption patterns that highlight the unique chemical composition and potential bioactive compounds in each solvent extract.

The FTIR spectrum of the methanol extract in Figure 2A indicates the presence of a peak at 3400 cm^−1^, while the peak at 3000 cm^−1^ signifies the presence of alcohol. Another peak at 2850 corresponds to the presence of an alkane (C-H). The peaks at 1650 and 1500 indicate the presence of (N-H), a secondary amine. Other peaks at 1150 and 1030 cm^−1^ correspond to the presence of (C-F), indicating aliphatic fluoro compounds. The FTIR spectrum of the n-hexane extract in Figure 2B shows different peaks. The absorption peaks at 3400 cm^−1^ suggest the existence of an O-H alcohol. Another peak 1650 cm^−1^ corresponds to the presence of a secondary amine. The peak at 1020 cm^−1^ represents the C-F aliphatic fluoro compounds.

Similarly, in Figure 2C, the ethyl acetate extract indicates the presence of stretchy peaks at 2900 and 2800 cm^−1^, corresponding to the presence of an alkane. The presence of an ester bond C = O was indicated by the peak that was seen at 1750 cm^−1^. Other peaks at 1150, 1130, and 1050 indicate the presence of aliphatic fluoro compounds. The peak at 550 represents the halogen compounds. The data was input into Origin 8.5 for the visualization of the different peaks in FTIR analysis. The major peaks observed were indicative of alkanes, alkyl halides, amines, and aldehydes, with significant values recorded at 781.17, 875.68, 923.90, 1018.41, 1240.23, 1319.31, 1361.74, 2357.01, 2850.79, and 2920.23.

### 3.6. GC-MS (Gas Chromatography-Mass Spectrometry) Analysis of n-Hexane, Ethyl Acetate, and Methanol Extracts of R. communis

#### 3.6.1. GC-MS Analysis of Ethyl Acetate Extract of *R. communis*

**Figure 3:**
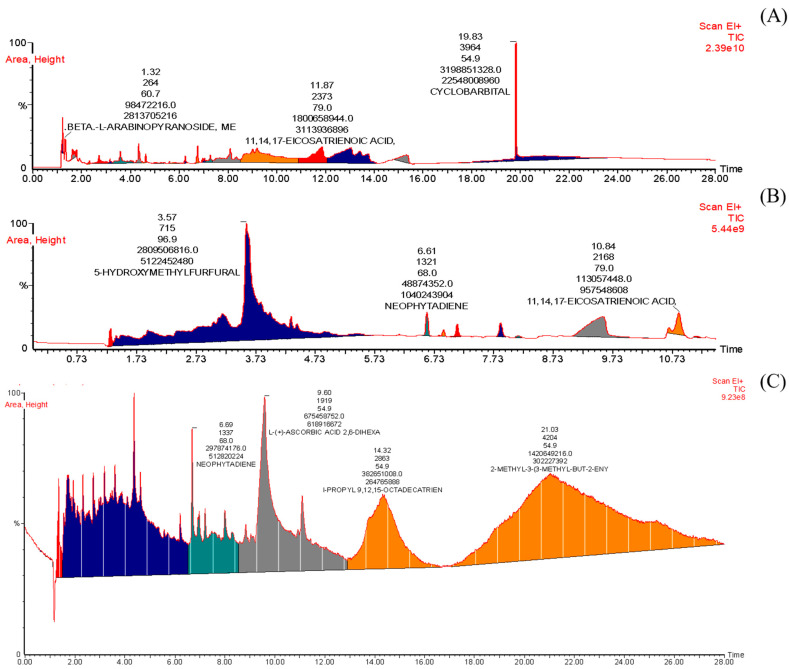
GC-MS analysis of ethyl acetate (**A**), methanol (**B**), and n-hexane (**C**) extracts of *R. communis*.

Figure 3A and Table 4 show the GC-MS analysis of the ethyl acetate extracts of *R. communis*. It can be seen that there are three distinct peaks representing some bioactive compounds in the extract. Each peak corresponds to a particular compound. The peak number 1, with a retention time of 1.32, corresponds to the beta-l-arabinopyranoside. Similarly, another peak eluting at a retention time of 11.87 corresponds to another compound, 11,14,17-eicosatrienoicacid. Peak number 3, with a retention time of 19.83, corresponds to another compound, which is cyclobarbital according to the GC-MS Turbo mass 5.4 online library.

#### 3.6.2. GC-MS Analysis of Methanol Extract of *R. communis*

Figure 3B and Table 4 depict the GCMS analysis of the methanol extract of *R. communis*. It can be seen that there are seven distinct peaks representing some bioactive compounds in the extract. Each peak corresponds to a particular compound. The peak number 1, with a retention time of 3.57, corresponds to 5-hydroxymethylfurfural. Similarly, another peak eluting with a retention time of 6.61 corresponds to another compound, neophytadiene. Peak number 3, with a retention time of 10.84, corresponds to another compound, which is 11,14,17-eicosatrienoic acid according to the GC-MS Turbo mass 5.4 online library.

#### 3.6.3. GC-MS Analysis of n-Hexane Extract of *R. communis*

Figure 3C and Table 4 show the GC-MS analysis of the n-Hexane extract of *R. communis*. It can be seen that there are four distinct peaks representing some bioactive compounds in the extract. Each peak corresponds to a particular compound. The peak number 1, with a retention time of 6.69, corresponds to neophytadiene. Similarly, another peak eluting with a retention time of 9.60 corresponds to l-(+)-ascorbic acid 2,6-dihexa. Peak number 3, with a retention time of 14.32, corresponds to 1-propyl 9,12,15-octadecatrien. Likewise, in peak number 4, with a retention time of 21.03, corresponds to another compound, which is 2-methyl-3(3-methyl-but-2-eny) according to the GC-MS Turbo mass 5.4 online library.

The following primary bioactive compounds, neophytadiene, l-(+)-ascorbic acid 2,6-dihexa, 1-propyl 9,12,15-octadecatrien, and 2-methyl-3(3-methyl-but-2-eny), are present in the n-hexane extract. The data was input into the Turbo Mass 5.4 software, and this software assigned names to these compounds. The details of these compounds are given in Table 4.

### 3.7. Multivariate Analysis

A total of 22 compounds were found in the ethyl acetate extract, and 13 compounds each were found in the methanol and n-hexane extracts (Figure 3). Neophytadiene was detected in all three extracts. Meanwhile, 11,14,17 eicosatrienoic acid and trans-cis, 1,8-dimethylspiro [4 were present only in the methanol and ethyl acetate extracts. According to HCA the ethyl acetate and n-hexane extracts showed similarity. Although the ethyl acetate is polar while n-hexane is non-polar solvent, yet the extraction chemistry of these solvent was quite similar. The clustering for the organic compounds indicated the similar behavior of 11,14,17 eicosatrienoic acid and trans-cis, 1,8-dimethylspiro [4. As these two compounds were present in the extracts of both methanol and ethyl acetate. As these two solvents are polar therefore, the 11,14,17 eicosatrienoic acid and trans-cis, 1,8-dimethylspiro [4 are the polar compounds.

**Figure 4:**
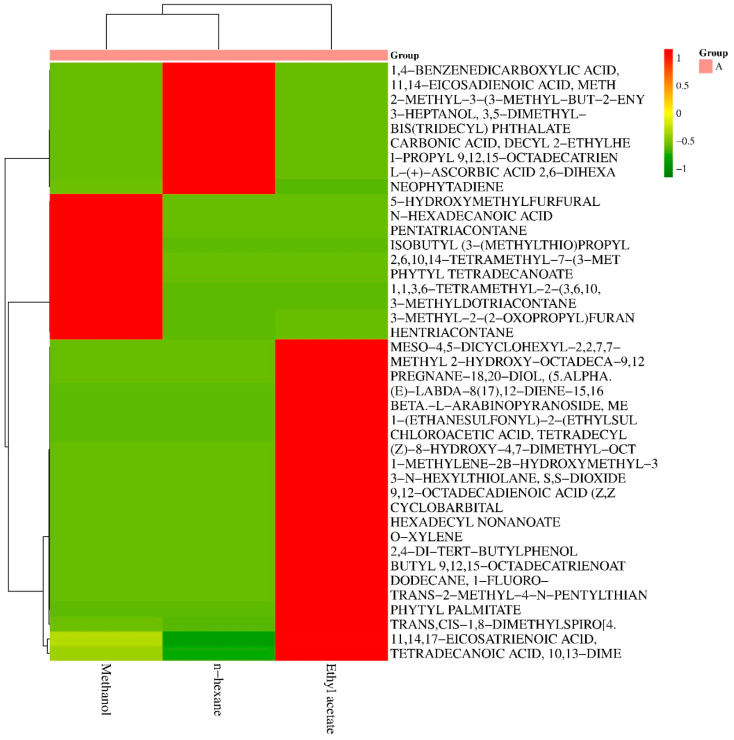
Cluster analysis of methanol, ethyl acetate, and n-hexane extracts of *R. communis*.

In cluster analysis Figure 4, various bioactive compounds from methanol, ethyl acetate, and n-hexane extracts from *R. communis* were classified into groups. In Table 5, 3-methyl-2-(2-oxopropyl)furan and hentriacontane were detected in methanol, ethyl acetate, and n-hexane extracts. Similarly, tetradecanoic acid 10,13-dime was detected in methanol and ethyl acetate extracts. Among all the above-mentioned compounds, 11,14,17-eicosatrienoic Acid and trans-cis-1,8-dimethylspiro [4] were highly significant based on linear regression analysis with a probability value less than 0.01. Comparing these two compounds based on the LC_50_ value, 11,14,17-eicosatrienoic acid exhibited effectiveness against *B. zonata*, with an LC_50_ value of 1.36.

## 4. Discussion

Management of *B. zonata* is very challenging for researchers and pest managers. The demand for horticultural crops, especially fruits, free from the infestation of fruit flies is on rise around the world [32]. There is no doubt that the effectiveness and regular misuse of synthetic insecticides causes different issues including insect resistance and environmental hazards or contamination [33]. Due to these problems, the world is currently turning towards biopesticides, which are environmentally friendly, easily available, and effective against insect pests [12] Although numerous control measures are in practice, there is always a demand for eco-friendly and effective management. In our study, we tested *R. communis* in different solvents, which exhibited insecticidal activity against *B. zonata* adults. This result might be due to the chemical constituents in the tested botanicals, which likely played a major role in killing the insects [34].

In the present results, 100% mortality was observed at a concentration of 2% in the n-hexane fraction of the crude extract of *R. communis*. Our findings are consisted with those of [35], who reported that when applied topically and to food, the n-hexane fraction of *R. communis* fruits and seeds at a 2% concentration had an ovicidal effect on *S. cosmioides* and *Spodoptera frugiperda*, significantly reducing larvae hatching and presenting with an insecticidal effect for the four instars of the three insect species. According to the present results, the *R. communis*-based biopesticides showed significantly different mortality effects on *B. cucurbitae* at different concentrations and time intervals from 24 to 72 h. Our findings are similar to previous findings [36], who observed that the *Euphorbia hirta* leaf extract was greatly effective against *Anopheles stephensi*. The percentage larval density reductions were increased with increasing time intervals from 24 to 72 h. In another study, Khoshraftar et al. [37] evaluated the biocidal efficacy of *Melia azedarach* extract-loaded nanoliposomes against *Myzus persicae* and *Trileurodes vaporariorum* pests. The maximum mortality rate in these pest species was found with high exposure times and concentrations. Similarly, Campolo et al. [38] observed the percentage mean mortality with Lemon, Mandarin, and sweet orange essential oils and their nanoparticles against *Tuta absoluta*. Both concentration and time-dependent increases in the mean mortality values were observed.

Our findings showed that the n-hexane extracts of *R. communis* were effective against *B. zonata*, providing better results and causing the highest mortality after 72 h. The results also showed that the leaves were the most potent in causing a high mortality rate. Our findings are similar to previous findings in [39], which reported that hexanic, acetonic, and methanolic extracts of *R. communis* leaves, fruits, and roots have insecticidal effects (Euphorbiaceae). These were used to control the succinic acid of apterous *Melaaphis sacchari* adults in contact bioassays at varying concentrations. With 96% mortality at 72 h, he discovered that the lower polarity chemical components in the hexane extract of *R. communis* leaves produced the best biological effect. In recent research, we isolated secondary metabolites, including fatty acids, diterpenes, and glycosides, from the crude extract of *R. communis* leaves. Our findings are consistent with those of [40], who indicated that *R. communis* contains a wide range of bioactive compounds, such as alkaloids, flavonoids, terpenoids, tannins, and glycosides. These compounds exhibit anticancer, insecticidal, antioxidant, antimicrobial, antinociceptive, and antidiabetic properties. The toxicity to test insects is due to these compounds, which are present in the selected plant.

We isolated four bioactive compounds in the n-hexane extract of *R. communis*, i.e., neophytadiene, l-(+)-ascorbic acid 2,6-dihexa, 1-propyl 9,12,15-octadecatrien, and 2-methyl-3(3-methyl-but-2-eny). The present results corroborated with the previous reports from [41], who showed that neophytadiene, an enzyme inhibitor belonging to the sesquiterpene class, stands as a key compound within marigold, playing a significant role in its insect repellent properties against pests. Additionally, ref. [42] also reported that the six main compounds that were detected in the n-hexane extract of *R. communis* were isophytol, n-hexadecanoicacid, 9,12,15-octadecatrienoic acid, oleic acid, octadecanoic acid, and tributylacetylcitrate. Our findings are similar to the previous results from [43], who checked the insecticidal activities of methanol, n-hexane, and ethyl acetate extracts from the seeds and leaves of *R. communis*, castor oil, and ricinine, with varying concentrations, against *Spodoptera frugiperda*. They determined that *R. communis* exhibited a better insecticidal activity against *Spodoptera frugiperda*, which justifies our results.

In our investigation of the n-hexane extract from the leaves of *R. communis*, we identified the presence of a specific fatty acid, 1-propyl 9, 12,15-octadecatrienoic acid. In contrast, previous research conducted by [43] revealed different fatty acids, specifically linoleic acid and linolenic acid, in the n-hexane leaf extract of *R. communis*. These fatty acids were found to exhibit insecticidal potential against *Spodoptera frugiperda*. This disparity in the chemical composition of the extracts underscores the importance of recognizing the diverse nature of plant extracts and its potential implications for their insecticidal properties. We also isolated several bioactive compounds from the methanol extracts of *R. communis*, which were found to be effective against *B. zonata*. Notably, three bioactive compounds were identified, including 5-hydroxymethylfurfural, neophytadiene, and 11,14,17-eicosatrienoic acid. The same compounds were also observed by earlier researchers [44], who found compounds that included n-methoxy-n-methylacetamide, glycerine, 4h-pyran-4-one, 2,3-dihydro-3,5-dihydroxy, 3-allyl-6-methoxyphenol, neophytadiene, 2,3a-dimethylhexahydrobenzofuran-7a, methyl ester, hexadecanoic acid, n-hexadecanoic acid, ricinine, 9,12,15-octadecatrienoic acid, phytol, methyl ester, cis,cis-7,10,13-hexadecatrienal, hexadecanoic acid, and lupeol. Concurrently, other researchers [45] have been investigating the potential of plant extracts, such as *Cascabela peruviana*, for their anti-insect properties. One study on *C. peruviana*, which used the fruit fly (*Drosophila melanogaster*) as a model, revealed that its ethanol extract, particularly derived from the stem and leaves, was effective against insects and contained polyphenol and flavonoid compounds. This study showed that *C. peruviana* extract induced mortality in 2nd instar larvae, disrupted the growth and reproduction of fruit flies, and even impacted the development of subsequent fruit fly generations. Another study, Waris et al. [46], revealed that the *R. communis* leaf extract contained components that were toxic to mosquito larvae.

Remarkably, our findings align closely with those from [47], who previously evaluated the efficacy of crude extracts from the castor plant *R. communis* against *Musca domestica* using dipping and thin film techniques. In both techniques, the laboratory bioassays yielded significant mortalities, underscoring the toxicity of the plant extract against the fly. Furthermore, the application of these extracts led to the occurrence of developmental aberrations, including reduced pupation rates and the failure of adults to emerge. These outcomes strongly suggest that the plant extracts contain active compounds that disrupt the hormonal control of development, thereby affecting the fly’s life cycle. It is concluded that plant phytochemicals can influence the biology and behavior of specific insect pests, exhibiting insecticidal, larvicidal, and ovicidal properties. These effects are notably observed within mostly each plant family when tested against various insect pests.

## 5. Conclusions

The present work is an attempt to add something new and useful through the use of *R. communis* leaves to control the *B. zonata*. This study concludes that the one bioactive compound 11,14,17 Eicosatrienoic acid that was detected in both methanol and in ethyl acetate extracts was the most effective against *B. zonata.* The GC-MS analysis reveals the presence of various bioactive compounds with insecticidal properties in the extract of leaves of *R. communis*, suggesting the potential of *R. communis* as a cost-effective and environmentally friendly agent for the management of *B. zonata*. Considering their affordability and biodegradability, these plant extracts could serve as alternatives to synthetic pesticides.

## Figures and Tables

**Table 1:** Toxicological effects of *R. communis* ethyl acetate extracts against *B. zonata* after 24, 48, and 72 h exposure periods.

Time	Concentration	Mortality (%)	LC_50_ (%)	LC_90_ (%)	F	*p*	Slope
24 h	0.5%	13.00 c	5.027 (3.39–24.37)	27.46 (27.46–10.05)	6.67	0.014	1.19 + 0.45
	1.0%	20.00 bc					
	1.5%	34.00 ab					
	2.0%	40.00 a					
	Control	0.00 ns					
LSD (0.05) for concentration = 15.373							
48 h	0.5%	22.00 b	4.204 (2.56–5.33)	20.41 (11.94–26.77)	6.05	0.0187	0.81 + 0.38
	1.0%	29.00 b					
	1.5%	35.00 ab					
	2.0%	50.00 a					
	Control	6.00 ns					
LSD (0.05) for concentration = 16.078							
72 h	0.5%	30.00 c	1.60 (1.60–0.73)	10.31 (5.27–20.00)	10.03	0.0040	0.75 + 0.33
	1.0%	45.00 bc					
	1.5%	53.33 b					
	2.0%	77.00 a					
	Control	13.00 ns					
LSD (0.05) for concentration = 19.78							

Lethal concentrations (LCs) are accompanied by 95% confidence limits (CLs) or LC90 of plant extract. Different lowercase letters indicate that the means are significantly different from each other at *p* = 0.05 and ns for non significant.

**Table 2:** Toxicological effects of *R. communis* n-hexane extracts against *B. zonata* after 24, 48 and 72 h exposure periods.

Time	Concentration	Mortality (%)	LC_50_ (%)	LC_90_ (%)	F	*p*	Slope
24 h	0.5%	20.00 b	5.72 (3.29–324.74)	67.09 (13.16–15.68)	3.89	0.0553	1.38 + 0.45
	1.0%	27.00 b					
	1.5%	34.00 ab					
	2.0%	48.00 a					
	Control	0.00 ns					
LSD(0.05) for concentration = 18.828							
48 h	0.5%	40.00 b	2.01 (0.01–6.14)	34.80 (8.52–45.79)	3.77	0.0593	1.08 + 0.34
	1.0%	47.00 b					
	1.5%	53.00 ab					
	2.0%	66.00 a					
	Control	0.00 ns					
LSD(0.05) for concentration = 19.185							
72 h	0.5%	53.00 b	0.94 (0.12–1.47)	6.75 (3.82–124.48)	4.33	0.0432	0.42 + 0.31
	1.0%	66.00 ab					
	1.5%	73.00 ab					
	2.0%	86.60 a					
	Control	13.00 ns					
LSD(0.05) for concentration = 21.961							

Lethal concentrations (LCs) are accompanied by 95% confidence limits (CLs). Different lowercase letters indicate that the means are significantly different from each other at *p* = 0.05 and ns for non-significant.

**Table 3:** Toxicological effects of *R. communis* methanol extracts against *B. zonata* after 24, 48, and 72 h exposure periods.

Time	Concentration	Mortality (%)	LC_50_ (%)	LC_90_ (%)	F	*p*	Slope
24 h	0.5%	20.00 b	5.72 (3.29–234.15)	67.09 (13.16–15.68)	3.89	0.0553	1.10 + 0.44
	1.0%	27.00 b					
	1.5%	33.00 ab					
	2.0%	46.00 a					
	Control	0.00 ns					
LSD(0.05) for concentration = 18.828							
48 h	0.5%	31.00 c	2.94 (1.64–4.26)	48.82 (10.29–42.78)	8.24	0.0079	0.80 + 0.38
	1.0%	38.00 bc					
	1.5%	45.00 b					
	2.0%	58.00 a					
	Control	0.00 ns					
LSD(0.05) for concentration = 13.381							
72 h	0.5%	38.00 b	1.38 (0.00–2.27)	22.80 (6.82–26.91)	4.00	0.0519	0.889 + 0.35
	1.0%	46.00 b					
	1.5%	53.00 ab					
	2.0%	67.00 a					
	Control	13.00 ns					
LSD(0.05) for concentration = 20.697							

Lethal concentrations (LCs) are accompanied by 95% confidence limits (CLs). Different lowercase letters indicate that the means are significantly different from each other at *p* = 0.05 and ns for non-significant.

**Table 4:** GC-MS analysis of n-hexane, ethyl acetate, and methanol extracts.

Types of Solvents	Name of Compounds	Class	% Composition	Retention Time	Function of Compounds
ethyl acetate	Neophytadiene	Diterpenes	1.26%	6.75	Neophytadiene is a natural organic compound belonging to the class of compounds known as diterpenes. Neophytadiene may have antioxidant properties and could play an important role in the defense mechanisms of plants against environmental stressors [18].
	Beta-L-Arabinopyranoside	Glycosides	0.63%	1.32	Beta-L-arabinopyranoside is a chemical compound that belongs to the group of arabinosides. These types of compounds are found in various natural sources, including plants and microorganisms. Some glycosides play essential roles in the bioactivity of various natural compounds, such as flavonoids, alkaloids, and other secondary metabolites. These compounds can have antioxidant, anti-inflammatory, or other biological effects [19].
	11,14,17-Eicosatrienoicacid	Fatty Acid	11.51%	11.87	11,14,17-Eicosatrienoic acid, often referred to as 11,14,17-ETA, is a member of the polyunsaturated fatty acid family. Eicosatrienoic acids have anti-inflammatory, anti-thrombotic, and anticancer properties. It also inhibits platelet aggregation [20,21].
	Cyclobarbital	Barbiturates	20.45%	19.83	Cyclobarbital belongs to the class of drugs known as barbiturates. Barbiturates function as depressants for the central nervous system and can produce sedative, hypnotic, and anesthetic effects [22].
	Trans-Cis, 1,8-Dimethylspiro [4]	Spiro compound	21.44%	9.19	Trans-cis, 1,8-dimethylspiro [4], also known as trans-cis-1,8-dimethylspiro[4,5]decane, exhibits antifungal, antimicrobial, and potential anticancer biological properties [23].
	3-Methyl-2-(2-Oxopropyl)Furan	Furan	0.05%	13.05	3-Methyl-2-(2-oxopropyl)furan, also known as mesifuran, exhibits antimicrobial, antioxidant, and anti-inflammatory biological properties [24].
Methanol	3-Methyl-2-(2-Oxopropyl)Furan	Furan	0.005%	16.92	3-Methyl-2-(2-oxopropyl)furan, also known as mesifuran, exhibits antimicrobial, antioxidant, and anti-inflammatory biological properties [24].
	N-Hexadecanoic Acid	Acid	9.77%	9.56	N-Hexadecanoic acid, also known as palmitic acid, exhibits antimicrobial, antioxidant, and anti-inflammatory biological properties [25].
	5-Hydroxymethylfurfural	Furan	81.79%	3.57	5-Hydroxymethylfurfural (5-HMF) is a chemical compound with the molecular formula C_6_H_6_O_3_ that is soluble in water and various organic solvents, including ethanol and methanol. 5-Hydroxymethylfurfural belongs to the furan family of organic compounds. The compound 5-HMF causes oxidative stress, disturbs glucose and lipid metabolism, and induces intestinal damage, damaging related signaling pathways, and ultimately affecting the development of various chemical reactions [26].
	Neophytadiene	Di terpenes	1.42%	6.61	Neophytadiene is a natural organic compound belonging to the class of compounds known as diterpenes. They kill the larvae of mosquitoes [18].
	3-Methyl-2-(2-Oxopropyl)Furan	Furan	0.09%	6.81	3-Methyl-2-(2-oxopropyl)furan, also known as mesifuran, exhibits antimicrobial, antioxidant, and anti-inflammatory biological properties [27].
	11,14,17-Eicosatrienoicacid	Fatty Acid	3.29%	10.84	The term “11,14,17-eicosatrienoic acid” refers to a type of fatty acid. Specifically, it is an omega-3 polyunsaturated fatty acid. They are known for their anti-inflammatory and cardioprotective properties. They have also larvicidal activity against *Culex quinquefasciatus* [21].
n-hexane	Neophytadiene	Di terpenes	7.53%	6.69	Neophytadiene may have antioxidant properties and could play an important role in the defense mechanisms of plants against environmental stress, in addition to having some larvicidal properties [18].
	L-(+)-Ascorbic Acid 2,6-Dihexa	Acid	17.07%	9.60	Dihexadecanoate is a term that refers to a compound derived from hexadecanoic acid (also called palmitic acid). It is a saturated fatty acid and belongs to the family of carboxylic acids. L-(+)-ascorbic acid dihexadecanoate has antibacterial, antitumor, and wound healing properties [28].
	I-Propyl 9,12,15-Octadecatrien	Polyunsaturated Fatty Acids	9.67%	14.32	“1-Propyl” suggests the presence of a propyl group attached to the compound. A propyl group consists of three carbon atoms (C_3_H_7_). “9,12,15-Octadecatrien” indicates the carbon atom positions and the number of double bonds in a long hydrocarbon chain. In this case, there are 18 carbon atoms (octadeca-) arranged in a chain with three double bonds (-triene). The numbers 9, 12, and 15 specify the positions of the double bonds within the chain. The biological roles of compounds related to linolenic acid often involve anti-inflammatory and antioxidant functions [29].
	Phenol,3,5-Bis(1,1-Dimethylethyl)-	Phenol	28.95%	4.36	Phenol, 3,5-bis(1,1-dimethylethyl)-, commonly known as 2,6-di-tert-butylphenol, is an organic compound with the chemical formula C_14_H_22_O. It exhibits antioxidant, antimicrobial, and anti-inflammatory biological properties [30].
	2-METHYL-3-(3-METHYL-BUT-2-ENY	isoprenes	35.91%	0.874	They have effective anti-insect activity against pupal and adult fruit flies [31].

**Table 5:** Cluster analysis of methanol, ethyl acetate, and n-hexane extracts of *R. communis*.

Name of the Compounds	R-Square	*p*-Value	LC_50_
Neophytadiene	0.21	0.005	0.09
11,14,17-Eicosatrienoic Acid	0.64 **	0.000	1.36
3-Methyl-2-(2-oxopropyl)Furan	0.04	0.264	1.6934
Hentricontane	0.04	0.232	0.0003
Trans-cis, 1,8-Dimethylspiro [4	0.60 **	0.000	4.273
Tetradecanoic acid 10,13,Dime	0.23 *	0.017	0.0234

* Significant (*p* < 0.5); ** highly significant (*p* < 0.01).

## Data Availability

All data pertinent to this work are presented in the paper. Any requests should be directed to the corresponding author.
